# Development of a point-of-care genetic test for effective treatment of ischaemic stroke: an early model-based cost-effectiveness analysis

**DOI:** 10.12688/wellcomeopenres.19202.1

**Published:** 2023-04-24

**Authors:** Stuart Wright, John McDermott, Dwaipayan Sen, Craig Smith, William Newman, Katherine Payne

**Affiliations:** 1 Manchester Centre for Health Economics, The University of Manchester, Manchester, M139PL, UK; 2 Manchester Centre for Genomic Medicine, The University of Manchester, Manchester, M13 9PL, UK; 3 Division of Evolution, Infection and Genomics, The University of Manchester, Manchester, M13 9PL, UK; 4 Greater Manchester Comprehensive Stroke Centre, Geoffrey Jefferson Brain Research Centre, Salford Royal Foundation Trust, Salford, M6 8HD, UK; 5 Division of Cardiovascular Sciences, Lydia Becker Institute of Immunology and Inflammation, Faculty of Biology, Medicine and Health, The University of Manchester, Manchester, England, M13 9PL, UK

**Keywords:** Pharmacogenetics, Stroke, Clopidogrel, Precision medicine, Economic evaluation, cost-effectiveness analysis, early health technology assessment.

## Abstract

**Background:** People who have experienced a stroke are at high risk of recurrent strokes. Clopidogrel is prescribed to people who have had a non-cardioembolic stroke. There is evidence that clopidogrel is not effective for patients with *CYP2C19* loss-of-function alleles. Pharmacogenetic testing is a potential strategy to identify such patients and guide prescription of appropriate antiplatelet treatment. This study aimed to provide an early estimate of the cost-effectiveness of using a point-of-care pharmacogenetic *CYP2C19* test in the UK National Health System.

**Methods:** A decision-analytic model comprising a linked decision tree and Markov model were created in R comparing pharmacogenetic testing with current prescribing practice. In the pharmacogenetic testing arm, patients identified to have one of three loss-of-function alleles were prescribed modified-release dipyridamole and aspirin or aspirin alone. Indicative data were sourced from reviews of the literature supported by expert consultation to select the most appropriate value for the input parameters. The healthcare costs (£;2021) and quality adjusted life years resulting from each strategy were estimated and the incremental cost-effectiveness of testing calculated. Deterministic threshold analysis and probabilistic sensitivity analysis (PSA) was conducted to account for uncertainty in the parameter estimates.

**Results:** The pharmacogenetic testing strategy generated 0.107 additional QALYs per patient tested and saved £512. Pharmacogenetic testing dominated current prescribing practice. The results were robust to extreme changes in key input variables. The PSA suggested that there was a 77% chance that pharmacogenetic testing would be cost-effective with a 62% chance it is cost-saving.

**Conclusions:** A point-of-care pharmacogenetic test to guide prescription of clopidogrel for people who have experienced a stroke has the potential to provide a significant health gain by preventing secondary strokes and may save resources in the health system. This early economic analysis has also informed the direction for future research.

## Introduction

In the United Kingdom there are over 100,000 hospital admissions for strokes annually and over a million people alive who have previously suffered at least one stroke ^1, 2^. People who have suffered a stroke have a shorter life expectancy and experience a lower quality of life, than people who have not suffered strokes ^3, 4^. Stroke survivors also have a high risk of experiencing subsequent strokes contributing to negative impacts on mortality and morbidity ^5^.

To reduce the risk of secondary stroke, people who have had an ischaemic, non-cardioembolic stroke (hereafter shortened to ‘stroke’), caused by a thrombus in one of the cerebral arteries, are prescribed anti-platelet medications to prevent further ischaemic events ^6^. Current guidelines by the National Institute for Health and Care Excellence (NICE) recommend the use of 300mg of aspirin daily for 14 days followed by 75mg clopidogrel daily as the first-line treatment. If clopidogrel cannot be tolerated then modified release-dipyridamole, aspirin, or a combination of these medicines can be used depending on which option is best tolerated ^7^.

Clopidogrel is a prodrug and is metabolised to its active form by an enzyme encoded by the *CYP2C19* gene. In the UK, 20-30% of individuals have one or two loss-of-function (LoF) alleles which result in the poor metabolism of medicines such as clopidogrel which, in turn, lowers the antiplatelet effect of the medicine. A potential consequence of ineffective clopidogrel is an increased risk of a secondary stroke. A systematic review conducted by Pan *et al.* (2017) suggested that people with LoF alleles (*2, *3, and *8) receiving clopidogrel had a risk of secondary stroke approximately double to individuals without the LoF alleles (Risk ratio: 1.92, 12.0% vs 5.8%) ^8^. Patients with the LoF alleles also had a higher risk of adverse vascular events compared with non-carriers ^8^.

If a patient’s *CYP2C19* genotype were known prior to prescription, those carrying LoF alleles could be prescribed an alternative anti-platelet medication if required, such as modified-release dipyridamole or aspirin. Using a genetic test to tailor the prescription of medicines to patients is known as pharmacogenetics ^9^. The goal of pharmacogenetic testing is to improve outcomes and make the best use of healthcare budgets by providing additional information for clinicians to select medicines that are effective with a lower risk of side effects ^10^. 

A prototype point-of-care test to detect *CYP2C19* LoF allleles has recently been developed by Genedrive PLC ^11^. A point-of-care tests enable a result to be delivered in a short timescale and/or near to the patient ^12^. The *CYP2C19* pharmacogenetic point-of-care test (hereafter called CYP2C19-test) uses a small platform which can be located within stroke units and produces results in around 40 minutes ^13^. This allows clinicians rapid access to genetic information to guide prescription of anti-platelet medicines in patients who have been admitted following a stroke.

Before implementing the *CYP2C19*-test in the NHS, it is important to determine the potential impact of changing the prescribing pathway on healthcare costs and consequences (benefits and harms) for patients. It is important to understand whether the total health benefits achieved for patients who have experienced a stroke outweigh the health benefits potentially lost by other patients in the health system when resources are allocated to the provision of the *CYP2C19-*test. The aim of this study was to conduct an early cost-effectiveness analysis of the use of the *CYP2C19*-test to guide prescription of clopidogrel, modified release dipyridamole, and aspirin. Early economic evaluations do not seek to provide definitive evidence as to whether an intervention is cost-effective or not but an indication as to whether it may be cost-effective and what areas of uncertainty may require further data collection to inform reimbursement decisions.

## Methods

This study used a decision-analytic model-based cost-effectiveness analysis with deterministic (one-way) and probabilistic sensitivity analysis. Table 1 provides an overview of the key aspects of the design of the analysis.

**Table 1.** Key design criteria.

Characteristic	Description
Decision problem	What are the incremental costs and consequences, and the degree of uncertainty in these estimates, of using a point-of-care *CYP2C19-*test to guide treatment of the secondary prevention of stroke?
Intervention	*CYP2C19* LoF alleles 2*, 3*, and 8* testing using point-of-care strategy in the hospital setting The results of the test are then used to guide treatment for the prevention of secondary stroke: - Patients with no LoF alleles receive clopidogrel. If clopidogrel not tolerated they can be switched to modified release dipyridamole and aspirin. Patients can also be switched to aspirin alone if modified release dipyridamole is not tolerated - Patients with one or more of the three LoF receive modified release dypridamole and aspirin. Patients can then be switched to aspirin alone if modified release dipyridamole is not tolerated. If dipyridamole is not tolerated then aspirin can be prescribed.
Comparator	No testing and all patients receive clopidogrel. If clopidogrel is not tolerated then the patient is switched to modified release dypridamole and aspirin. Patients can then be switched to aspirin alone if modified release dipyridamole is not tolerated
Population	A cohort of patients in the UK representing an estimate of the number of people who have their first stroke each year (n=71,091). The mean age of the cohort at the start of the analysis was set at 67 years. Data relevant to a European cohort of patients were used to populate the model.
Model types	Decision tree (to represent the testing or current prescribing pathways) linked with a state transition Markov model (to represent the treatment pathways)
Programming software	R version 3.6.1 ^17^
Time horizon	Lifetime
Cycle length	1 year
Discount rate	3.5% for costs and consequences
Perspective	NHS (health system)
Costs	UK £ in 2021 prices
Consequences	Quality Adjusted Life Years (QALYs) Number of non-fatal strokes prevented Number of fatal strokes prevented
Uncertainty	Deterministic threshold analysis Probabilistic sensitivity analysis
Cost-effectiveness threshold	£20,000 per QALY In accordance with NICE Reference Case ^a^

^a^ NICE. Methods guide for technology appraisal 2013 ^18^.

### Model conceptualisation

The first stage in structuring the decision-analytic model that formed the basis for the cost-effectiveness analysis was to establish how the proposed *CYP2C19-*test and treatment pathways with subsequent events compared with current prescribing should be represented. This model conceptualisation was completed in line with published recommendations ^14^. Several important decisions and assumptions were made during the model conceptualisation process to focus the cost-effectiveness analysis of *CYP2C19-*test for secondary prevention of stroke informed by a systematic review and expert input.

The systematic review used an electronic search of two databases (Embase: from inception to December, 2019; MEDLINE: from inception to December, 2019). A search strategy was developed combining search terms for clopidogrel, genotyping, and stroke (taken from (7)) with a published electronic search strategy comprising terms to identify economic evaluations ^15^. After de-duplication this electronic search yielded 119 results. None of the identified studies met the inclusion criteria of having a primary focus on stroke patients. This result matches the findings of a published systematic review of economic evaluations of pharmacogenomics technologies in cardiovascular disease which identified 16 studies investigating *CYP2C19* genotyping in acute coronary syndrome, but none in patients experiencing a stroke ^16^.

In the absence of any existing published studies on *CYP2C19* pharmacogenetic testing, a bespoke model structure was conceptualised, informed using expert input (two clinical geneticists and two consultants specialising in the treatment of stroke). An existing decision-analytic model-based cost-effectiveness analysis produced as part of a NICE technology appraisal of clopidogrel was used to inform the selection of relevant health states for people experiencing a stroke ^19^. The model conceptualisation process focused on defining: the relevant jurisdiction; study perspective; time horizon; relevant pathways.

- *The Relevant Jurisdiction:* The UK NHS.
- *Study Perspective:* the analysis assumed a relevant health care service perspective because the analysis aimed to inform how to spend the NHS budget. In accordance with this perspective only items of resource use and associated costs accruing to the health services were included. Costs to individual patients were excluded from the analysis.
- *Time horizon*: a life-time horizon was assumed because patients who have had an initial stroke remain at risk of subsequent strokes for the remainder of their lives.
- *Relevant pathways:* the model needed to capture the initial testing pathways (CYP2C19- testing or current prescribing) to identify which treatment pathway is relevant for a patient and then follow subsequent events that may occur including up to death from stroke or other causes (see Table 1).

### Model Structure

**Figure 1.**
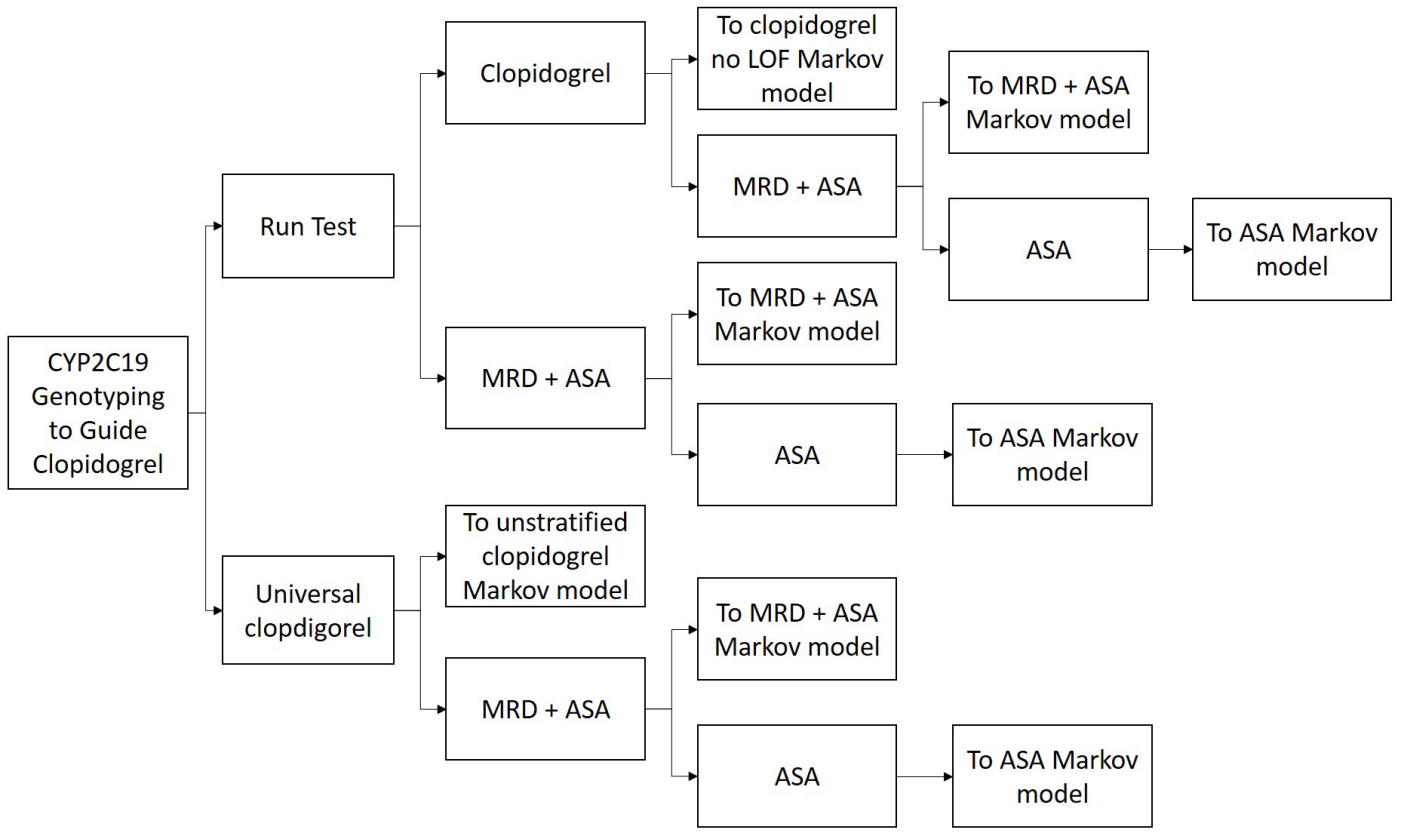
Decision tree of the *CYP2C19* testing component.

**Figure 2.**
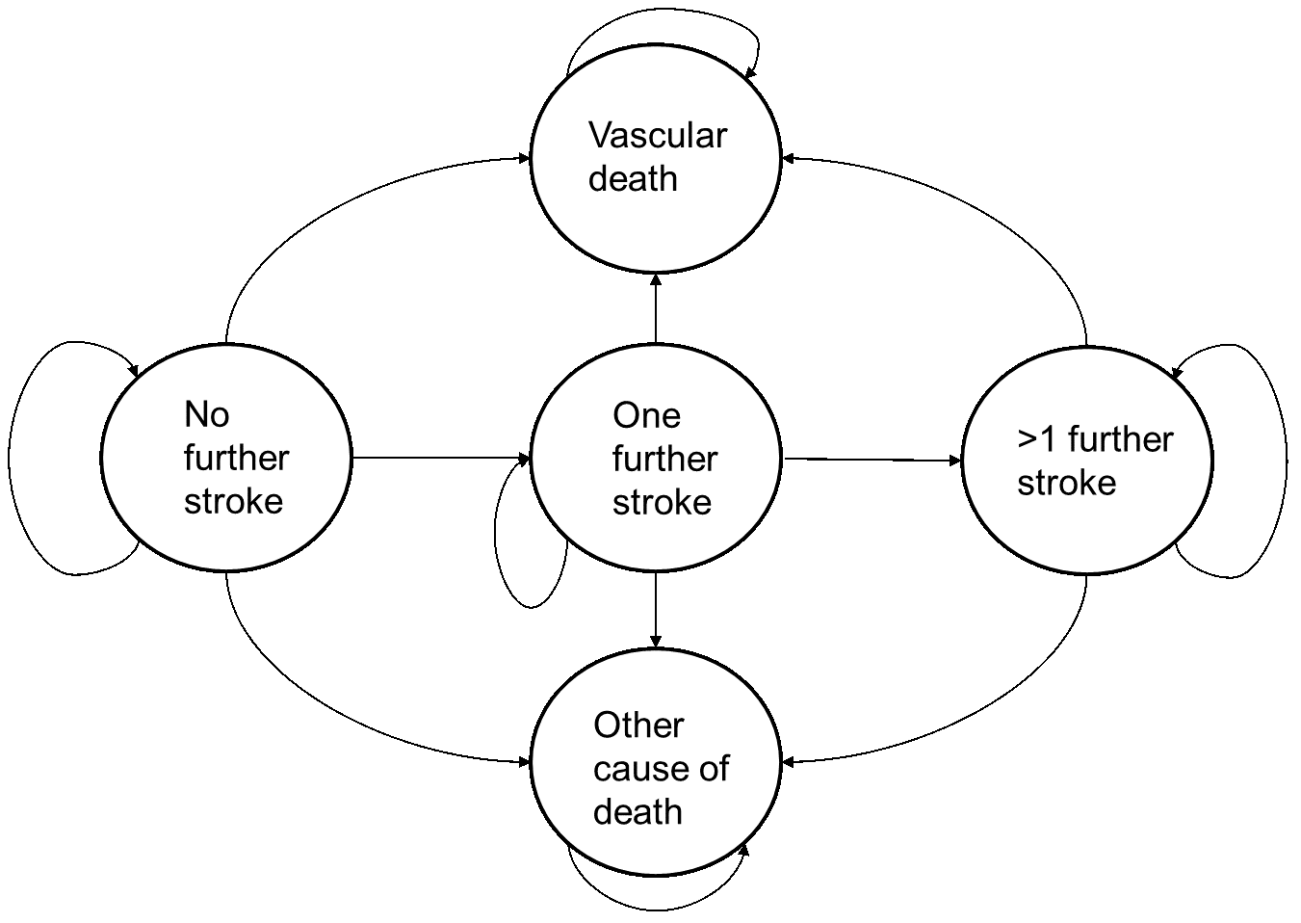
State transition Markov model.

The conceptualised model was structured using a decision tree to represent the testing or current prescribing pathways, linked with a state transition Markov model, for the treatment pathway and subsequent health events related to stroke. A bespoke decision tree was created (see Figure 1) to represent the *CYP2C19-*test process compared with no testing. The events associated with treatment type were captured using a stated transition Markov model (see Figure 2) with five health states: no additional strokes; one additional stroke; two or more additional strokes; vascular death; non-vascular death. Patients were assumed to enter the state transition Markov model in the ‘no additional stroke’ state. Patients then moved through the health-states based on a series of transition probabilities.

***Estimating the patient population.*** The population used in this study was an estimate of the number of people who experience a first ischaemic stroke in the UK in 2019. A report produced by Public Health England estimated that there were approximately 57,000 first time strokes in England in 2016 ^20^. In order to determine and estimate for the number of first time strokes in the whole of the UK in 2019, the first time stroke incidence for 2016 (1.07 per 1,000) identified in this report was applied to the Office for National Statistics population estimates from 2018 (66,436,000), the latest available year ^21^. This yielded an estimate of 71,087 first strokes in the UK in 2019. Of these, approximately 85% of strokes are classed as ischaemic, as opposed to haemorrhagic ^22^. This calculation estimated a final population size of 60,424 first-time ischaemic strokes in the UK in 2019. It was assumed that the mean age of people experiencing these strokes was 67 years in line with the assumption used for patients who had experienced an ischaemic stroke in NICE Technology Appraisal 90 ^19^. An assumption was made that all strokes included in this model would be treated as atherothrombotic (and treated with anticoagulants) as opposed to cardioembolic strokes (which would be treated with anticoagulants). 

### Populating the model

A process of sequential reviews of the literature supported by expert consultation was used to obtain the most appropriate value for the parameters. The process of populating the model is now described for each parameter needed for the decision tree and Markov model.

### Probabilities

The main route of action for the *CYP2C19*-test is to change the probability of having a subsequent stroke for patients receiving clopidogrel. In the general population estimates suggest approximately 75% of people of European ethnicity can metabolise clopidogrel into its active component while 25% cannot ^8^. The probability of a patient receiving clopidogrel having a stroke was calculated as the mean (average) probability of patients from either metaboliser status group having a stroke. The *CYP2C19-*test provides information to separate the population into two distinct groups of potential (i) responders and (ii) non-responders. Patients who receive clopidogrel and metabolise clopidogrel are classed as ‘responders’ are assumed to have a lower risk of stroke than the average patient in the population. Patients who do not metabolise clopidogrel are assumed to be non-responders and have a higher risk of stroke. These patients are instead prescribed modified release dipyridamole and/or aspirin. It is assumed that patients switched to modified release dypiridamole and/or aspirin have similar levels of secondary stroke to clopidogrel in the following the current prescribing pathway strategy. The assumed reduction in risk of patients with no LOF alleles having a stroke when receiving clopidogrel is taken from systematic review and meta-analysis by Pan *et al.,* 2017 ^8^. The proportion of patients with CYP2C19 loss-of-function alleles was also taken from this study

The transition probabilities used to populate the treatment component of the model (Markov model) were based on evidence synthesised from four trials in NICE technology appraisal 90 ^19^. Relevant transition probabilities included the likelihood of one or more additional strokes when patients were receiving each medicine and the likelihood of vascular death. All-cause mortality was included based on the Office of National Statistics (ONS) mortality data ^21^. The probability of patients experiencing the adverse events of minor bleeding events, major bleeding events, and worsening cardiac heart function were included based on estimates in the NICE Technology Appraisal 90 ^19^.

### Resource use and costs

Table 2 describes the relevant items of resource use and costs consistent with the selected study perspective. In each health state in the state transition Markov model patients were assigned a cost of treatment based on the severity of the health state. Patients were also assigned a one off cost when they had an additional stroke, died due to a stroke, or died due to other causes. It was necessary to use summary data from published sources ^19^. The primary data source was based on 2010 costs, these were inflated to 2021 prices using the hospital & community health service index (2010 to 2015) and its replacement, the NHS cost inflation index (2015 to 2021) ^23^.

**Table 2.** Resource use and unit costs.

Resource use	Unit cost (2021 £)	Source
Point of care test cost	£60	Manufacturer
Staff time to conduct the test	£16.91	Assumption based on expert opinion 10 minutes of nurse time and 5 minutes of consultant time. 26
Annual cost of treating non-disabling stroke	£2,087	19
Annual cost of treating disabling stroke	£6,407	19
One time cost of a non-disabling stroke	£7,936	19
One time cost of a disabling stroke	£16,896	19
One time cost of a fatal stroke	£10,855	19
One time cost of a non-stroke death	£2,755	19
Annual cost of treating adverse events associated with clopidogrel	£25	19
Annual cost of treating adverse events associated with modified- release dipyridamole and aspirin	£32	19
Annual cost of treating adverse events associated with aspirin	£27	19
Annual cost of clopidogrel	£32	27
Annual cost of modified-release dipyridamole and aspirin	£82	28
Annual cost of aspirin	£22	29

### Health consequences

In each health state in the state transition Markov model patients were assigned a health utility value to represent the severity of the health state. Utility values are a preference-based measure of health-related quality of life ^24^ and are needed to convert the estimated years of survival into a quality adjusted value to estimate quality-adjusted life-years (QALYs) by simple multiplication.

The utility values used by the evidence review group appraising the published NICE Technology Appraisal 90 report for the stroke health states were not publicly available. Therefore, the utility values used by Bristol-Myers Squibb in their submission to the NICE appraisal of clopidogrel were used. A utility weight of 0.61 was used for the no further stroke state. A decrement of 0.174 was applied to provide values for the one additional stroke and two or more additional strokes states (utility = 0.436). Disutilities associated with experiencing adverse events associated with each medicine were available from the evidence review group report. These were 0.0033 for a minor bleed, 0.1426 for a major bleed, and 0.0163 for worsening cardiac heart function.

### Data analysis

A base case analysis was used to calculate the total costs and QALYs for a sample of 60,424 patients over a lifetime from the pre-defined starting age of 67 years. An incremental analysis was performed. This process involves first calculating the total costs, life-years and QALYs for each pathway. The incremental cost per QALY gained was then calculated using the formula:


$$ICER=\frac{\left(C_{2}-C_{1}\right)}{\left(QALY_{2}-QALY_{1}\right)}\;(1)$$

Where *QALY*_2_ is the total QALYs of *CYP2C19-*testing pathway, *QALY*_1_ is the total QALYs for the current prescribing pathway, and *C*_2_ and *C*_1_ are the respective total costs for each pathway.

Net health and monetary benefit per patient and in total across the patient population were calculated ^25^. The net health benefit (NHB) and net monetary benefit (NMB) of the intervention represents the difference between the change in health for patients receiving the intervention and the change in health for patients in the health system resulting from a reallocation of resources to the intervention. The values of NHB and NMB are calculated using the following formulae


$$NHB=\text{Δ}QALY-\frac{\text{Δ}Cost}{\lambda }\;(2)$$

NHB=ΔQALY.λ−ΔCost(3)

Where Δ *QALY* is the incremental QALYs per patient arising from the use of the intervention, Δ *Cost* is the incremental cost per patient from the use of the intervention, and λ is the NICE cost-effectiveness threshold, which in this study was taken to be £20,000 per QALY. The total NHB and NMB of the intervention was calculated by multiplying these figures by the population size. If NHB or NMB are positive, then the introduction of the intervention will increase the total health produced by the health system.

Two types of sensitivity analysis were used to determine the impact of uncertainty on the results of the cost-effectiveness analysis: deterministic threshold analysis and probabilistic sensitivity analysis. In the threshold analysis, key parameters were varied one at a time until the intervention became cost-ineffective defined by exceeding a threshold of £20,000 per QALY gained. The maximum or minimum values for the risk ratio for patients receiving stratified clopidogrel, the *CYP2C19-*test cost, and the tolerance of clopidogrel and modified release dipyridamole were determined and reported.

To incorporate uncertainty in all of the input parameters simultaneously, a PSAs was conducted. Distributions were assigned to each parameter, representing the pattern of uncertainty around the point estimate (see appendix 1). The model was re-run across 10,000 iterations, with new parameter values being randomly drawn from the distributions in each iteration. For each iteration, the estimated incremental costs, benefits, ICERs, NHB, and NMB, were recorded. An incremental cost-effectiveness plane was created to illustrate the distribution of the incremental health costs and consequences. The probability that the intervention would be cost-effective given the parameter uncertainty was calculated by determining the proportion of iterations which exhibited a positive NHB and NMB.

## Results

This section first presents the results of the base case analysis and then each sensitivity analyses.

### Base case analysis

The deterministic base case analysis suggested that using the *CYP2C19-*test was £512 less expensive compared with no testing per patient and generated 0.107 additional QALYs (see Table 3). This indicates that *CYP2C19* testing was the dominant strategy as it was cheaper and more effective when compared with no testing. The testing strategy generated a NHB of 0.133 QALYs per patient tested or a NMB of £2,652 per patient. Across the assumed patient population of 60,424, this equates to a total NHB of 8,012 QALYs or a total NMB of £160,244,448.

**Table 3.** Estimated healthcare costs and consequences.

Intervention	Total costs (£; 2021)	Total QALYs	Incremental cost per QALY gained
*Deterministic base case analysis*			
CYP2C19 testing	£59,059	7.409	Not applicable *CYP2C19* testing dominant
No testing	£59,517	7.302	
*Probabilistic sensitivity analysis*			
CYP2C19 testing	£58,769	7.363	Not applicable *CYP2C19* testing dominant
No testing	£58,893	7.271	

### Probabilistic Sensitivity Analysis

Table 3 shows the results from the PSA. These closely match the estimates expected values from the base case analysis but slight differences are observed because the PSA returns the average (mean) values from a random sample of runs of the model. The probabilistic sensitivity analysis suggested that *CYP2C19* testing was £124 less expensive compared with no testing per patient and generated 0.092 additional QALYs ( Table 3). This indicates that *CYP2C19* testing was the dominant strategy as it was cheaper and more effective when compared with no testing. The testing strategy generated a net health benefit of 0.098 QALYs per patient and a net monetary benefit of £1,964 per patient. This equates to a total net health benefit of 5,934 QALYs and a total net monetary benefit of £118,672,736 for a population of 60,424.

**Figure 3.**
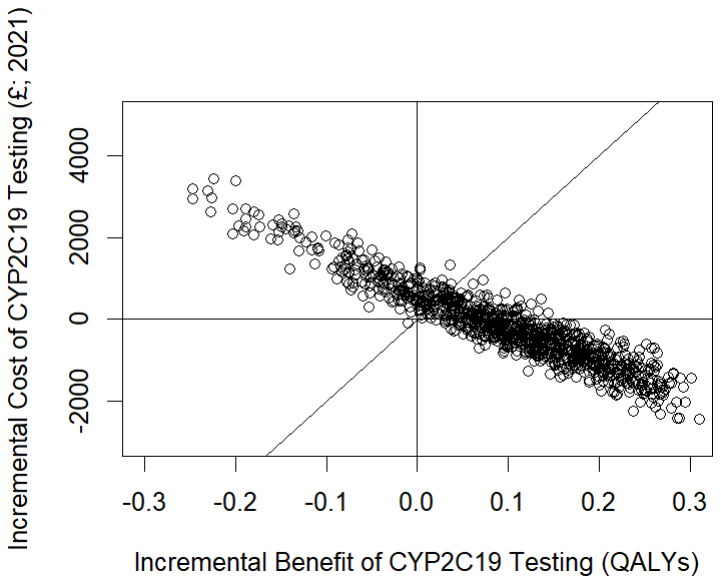
Cost-effectiveness plane showing the results of the probabilistic sensitivity analysis.

Figure 3 shows the plot of the probabilistic sensitivity analysis results. The analysis suggested that there is a 77% chance that the intervention is cost-effective, at a threshold of £20,000 per QALY, given the uncertainty in the parameter estimates. In 62% of the iterations in the probabilistic sensitivity analysis the intervention was cost saving to the health system.

### Threshold Analysis

The results of the cost-effectiveness analysis were robust to substantial changes in the parameters included in the threshold analysis. The risk ratio for the effectiveness of clopidogrel for patients with no loss-of-function compared with the un-stratified population could rise from 0.702 to as high as 0.94 before the intervention became cost-ineffective at a threshold of £20,000 per QALY. The cost of the *CYP2C19-*test could rise from £60 to £1,357 before it became cost-ineffective. Tolerance to taking clopidogrel could fall to as low as 4% and *CYP2C19-*testing remain cost-effective. Changing the assumed tolerance to taking modified-release dipyridamole did not impact the absolute cost-effectiveness of the intervention at any assigned level.

## Discussion

This study used an early model-based cost-effectiveness analysis to estimate the incremental costs and QALYs associated with introducing a *CYP2C19-*test to guide prescription of clopidogrel in patients who had experienced a stroke. The use of the *CYP2C19-*test was found to dominate current prescribing practice of universal clopidogrel prescription, improving patients’health and reducing costs to the health system. Sensitivity analysis confirmed that the intervention was highly likely to be cost-effective and likely to be cost saving even when accounting for uncertainty in the currently available evidence.

The results of this study suggest that the introduction of a *CYP2C19*-test could significantly reduce the number of secondary and tertiary strokes in the UK. When clopidogrel is offered universally, its clinical effectiveness is, on average, very similar to modified-release dipyridamole or aspirin. However, this observed effectiveness is a product of the mixing of two groups of people within a population; those with LoF alleles and those without. In this early analysis, patients without LoF alleles receiving clopidogrel had an annual risk of stroke of 2.79% while those with LoF alleles had a stroke risk of nearly 9%. Testing patients and switching the medicine for people with LoF alleles to modified-release dipyridamole or aspirin approximately halved their risk of having a stroke in the next year.

Studies investigating the cost-effectiveness of *CYP2C19* testing have been published but these are not relevant to the UK setting. The results of these international studies echo those found in this early economic analysis. Studies using similar modelling strategies in China and Canada have shown that testing for *CYP2C19* variants is highly likely to be cost-effective in clinical practice, albeit it with less chance of saving money for the health system ^30, 31^. A study investigating the cost-effectiveness of testing in people of Asian heritage, where LoF alleles are more prevalent, found testing to be cost-effective when patients with LoF alleles were switched from clopidogrel to ticagrelor ^32^.

This study presented an early model-based cost-effectiveness analysis of *CYP2C19* testing to guide prescription of clopidogrel in the UK. Much of the key data used in this analysis was taken from the 2010 NICE appraisal of clopidogrel compared to modified-release dipyridamole and aspirin. This study may now be out of date and a full economic evaluation using an up-to-date search of the literature should be conducted to confirm the findings presented in this early analysis. In particular, it is likely that the cost of treating patients with stroke will have changed in the 12 years since this study due to changes in treatment and management pathways. However, the aim of this study was to create an early economic evaluation that could be further developed. The public availability of the model will allow researchers to update the input parameters with new information from trials of *CYP2C19* testing in order to address the sources of uncertainty in the model.

This study was limited in the focus on the consequences of *CYP2C19* testing on the prevention of further ischaemic strokes. However, the appropriate prescription of anti-platelet medications will also help reduce the risk of other cardiovascular diseases. As such, the benefits of testing may be underestimated. Furthermore, the model developed in this study only includes patients who suffered an ischemic stroke and did not capture those who had an index transient ischemic attack (TIA). The risk of developing an ischemic stroke following a TIA is significantly increased compared to control populations. As such effective secondary prevention is critical in this population and, as with ischemic stroke, clopidogrel is a commonly used agent. By not including patients with TIA in this model, the benefits of *CYP2C19* guided genotyping may have been underestimated. In addition, the *CYP2C19* gene is implicated in the metabolism of a range of other drugs including proton pump inhibitors and selective serotonin uptake inhibitors ^33, 34^. If the information from the test can be stored in a patient’s records for use by other clinicians in their prescribing decisions, then there would likely be additional health benefits at little additional cost. 

It should be noted that this model made a number of assumptions, including the fact that all index strokes were treated as atherosclerotic in origin and indicated antiplatelet therapy. In reality, a proportion of strokes will be cardioembolic in nature and require anticoagulant treatment. This granularity was not captured in this modelling exercise and should be considered when interpreting the findings. Finally, this model includes two main therapeutic strategies, clopidogrel and modified-release dipyridamole plus aspirin. Ticagrelor has been investigated in several recent studies for patients with ischaemic stroke, and particularly *CYP2C19* LoF allele carriers. If ticagrelor becomes used as part of routine practice, then this agent should be included in any future economic modelling ^35, 36^. 

## Conclusion

This early decision-analytic model-based analysis has produced indicative estimates of the relative cost-effectiveness of using a point-of-care *CYP2C19* test to guide the treatment of the secondary prevention of stroke. This early analysis suggested that using a point-of-care *CYP2C19* test to guide clopidogrel prescription would be likely to be cost-saving to the NHS. There was considerable uncertainty in this indicative analysis that used currently available evidence. There is a clear need for a definitive trial to evaluate the clinical and cost-effectiveness of using a point-of-care *CYP2C19* test to guide the treatment of the secondary prevention of stroke. 

## Data Availability

The data underpinning the economic analysis in this study were sourced from the following studies: Greenhalgh, J., Saborito, C. M., Bagust, A., Boland, A., Oyee, J., Blundell, M., Dundar, Y., Dickson, R., Proudlove, C., & Fisher, M. (2010). Clopidogrel and modified-release dipyridamole for the prevention of occlusive vascular events (review of Technology Appraisal No. 90). Pan, Y., Chen, W., Xu, Y., Yi, X., Han, Y., Yang, Q., Li, X., Huang, L., Johnston, S. C., Zhao, X., Liu, L., Zhang, Q., Wang, G., Wang, Y., & Wang, Y. (2017). Genetic Polymorphisms and Clopidogrel Efficacy for Acute Ischemic Stroke or Transient Ischemic Attack: A Systematic Review and Meta-Analysis. Circulation, 135(1), 21–33. https://doi.org/10.1161/CIRCULATIONAHA.116.024913 National institute for Health and Care Excellence. (2022). Clopidogrel. British National Formulary. https://bnf.nice.org.uk/drugs/clopidogrel/ National institute for Health and Care Excellence. (2022). Clopidogrel. British National Formulary. https://bnf.nice.org.uk/drugs/clopidogrel/ National institute for Health and Care Excellence. (2022). Clopidogrel. British National Formulary. https://bnf.nice.org.uk/drugs/clopidogrel/ Office for National Statistics. (2019). Population estimates for the UK, England and Wales, Scotland and Northern Ireland - Office for National Statistics. https://www.ons.gov.uk/peoplepopulationandcommunity/populationandmigration/populationestimates/bulletins/annualmidyearpopulationestimates/mid2018 Curtis, L. A., & Burns, A. (2019). Unit Costs of Health and Social Care. https://doi.org/10.22024/UniKent%2F01.02.79286 Intercollegiate Stroke Working Party. (2016). National clinical guideline for stroke (5th Editio).

## Software availability

Zenodo. stuwrighthealthecon/CYP2C19: Version 1.0. DOI: 10.5281/zenodo.7708459

This project contains the following underlying data:

- CYP2C19.R (An R script used to conduct the deterministic analysis reported in this study)
- PSA CYP2C19.R (An R script used to conduct the probabilistic sensitivity analysis reported in this study)

The R scripts containing the code required to run the analyses reported in this paper are publicly available at https://github.com/stuwrighthealthecon/CYP2C19.

License: Data are available under the terms of the Creative Commons Zero "No rights reserved" data waiver (CC0 1.0 Public domain dedication).
