# Supplementary material for: Development of a point-of-care genetic test for effective treatment of ischaemic stroke: an early model-based cost-effectiveness analysis
Source: Wellcome Open Res. 2023 Apr 24;8:183. [Version 1] doi: 10.12688/wellcomeopenres.19202.1 (PMC10587658; doi:10.12688/wellcomeopenres.19202.1)
Supplement: Peer review history

# Reviewer report: Reviewer response for version 1

**DOI:** 10.21956/wellcomeopenres.21284.r66103

**Published:** 2023-10-19

**Authors:** Alex Doney

**Affiliations:** 1 University of Dundee, Dundee, UK

This report is a valuable contribution to further knowledge in this area. 

 Although there are a number of assumptions made in the modelling these are largely acknowledged in the discussion appropriately.  

 The model is also limited and rather overly simplistic in a number of ways such as using estimates from many years ago and only considering point of care testing and this reviewer is not convinced that the overall costs of point of care testing using the device under consideration have been fully accounted for. On the other hand as acknowledged by the authors the overall cost of recurrent stroke and indeed other events is underestimated in this model.

 The discussion should include acknowledgement of other potential ways of testing beyond point of care testing.

 The assumption of using all ischaemic strokes as opposed to removing the proportion with cardioembolism inflates the overall population and consequently all subsequent global saving estimates for pharmacogenetics testing. This should be corrected.

 In this context there is also an important typographical error in the last sentence of the paragraph "Estimating the patient population". ..."all strokes included in this model would be treated as atherothrombotic (and treated with anticoagulants)" - the authors mean to say "antiplatelets".

Is the work clearly and accurately presented and does it cite the current literature?

Yes

If applicable, is the statistical analysis and its interpretation appropriate?

Yes

Are all the source data underlying the results available to ensure full reproducibility?

Yes

Is the study design appropriate and is the work technically sound?

Yes

Are the conclusions drawn adequately supported by the results?

Yes

Are sufficient details of methods and analysis provided to allow replication by others?

Yes

Reviewer Expertise:

Pharmacogenomics and stroke medicine

I confirm that I have read this submission and believe that I have an appropriate level of expertise to confirm that it is of an acceptable scientific standard.

# Reviewer report: Reviewer response for version 1

**DOI:** 10.21956/wellcomeopenres.21284.r67774

**Published:** 2023-10-19

**Authors:** Kaavya Narasimhalu

**Affiliations:** 1 National Neuroscience Institute, Singapore, Singapore

In this study, the authors examine the cost effectiveness of point of care testing for three LOF alleles prior to the prescription of clopidogrel, with aspirin and dipyridamole or aspirin as alternative agents. This is an interesting study, but it can be improved if the comments below were addressed.

 Major comments:

1. It would be better for the authors to look at the LOF rates in the UK population that uses the NHS based on its current ethnic breakdown rather than to focus on those of European ancestry alone. More so considering the LOF rates are higher in non-European populations.
2. Part of the issue with dipryridamole as an alternative is that it is poorly tolerated compared to aspirin and clopidogrel. Is there data available about the rates of discontinuation of dipyridamole within stroke patients? Could that information be incorporated into your models?

Minor comments:

1. I think there is a mistake in the last sentence of the estimating the patient population section. Do the authors mean to say. “An assumption was made that all strokes included in this model would be treated as atherothrombotic (and treated with *antithrombotics*) as opposed to cardioembolic strokes (which would be treated with anticoagulants)."?
2. First paragraph of discussion section, I believe there is a space missing in between these words: "improving patients’health".

Is the work clearly and accurately presented and does it cite the current literature?

Yes

If applicable, is the statistical analysis and its interpretation appropriate?

Yes

Are all the source data underlying the results available to ensure full reproducibility?

Yes

Is the study design appropriate and is the work technically sound?

Yes

Are the conclusions drawn adequately supported by the results?

Yes

Are sufficient details of methods and analysis provided to allow replication by others?

Yes

Reviewer Expertise:

pharmacogenomics, neurology, including stroke

I confirm that I have read this submission and believe that I have an appropriate level of expertise to confirm that it is of an acceptable scientific standard, however I have significant reservations, as outlined above.

# Reviewer report: Reviewer response for version 1

**DOI:** 10.21956/wellcomeopenres.21284.r56556

**Published:** 2023-06-09

**Authors:** Nicky J. Welton, Joe Carroll

**Affiliations:** 1 Bristol Medical School (PHS), University of Bristol, Bristol, UK; 2 University of Bristol, Bristol, UK

This paper describes an early economic evaluation of a new point-of-care genetic test for targeted treatment to prevent further strokes in patients who have had an ischaemic stroke. Overall, this a well-written and useful article, although more detail is needed on the model/inputs. The model makes a lot of assumptions, which is to be expected from an early economic evaluation, but in which case we would also expect there to be more exploration of the sensitivity of the results to those assumptions. There was insufficient detail to know what the model inputs were, which may in part be because Appendix 1 was missing. Specific comments are given below:

1. The final sentence of the abstract is the first point it becomes clear in the abstract that this is an early economic analysis. This needs to be clearer earlier on in the abstract, as to the aim of the model (as you state in last sentence of the Intro).
2. Introduction 3 ^rd^ para: Give confidence interval alongside estimate of RR.
3. Intro 3 ^rd^ Para “dipyridamole or aspirin”: Clarify that this is in combination or if dipyridamole not tolerated then aspirin monotherapy. Same comment elsewhere where this is used.
4. Intro 5 ^th^ para: We recognise that the focus here is on the cost-effectiveness of GeneDrive, but Genomadix is also available as a point-of-care test, and that should at least be mentioned in the Intro and Discussion. Laboratory-based testing is also an option, and again should be mentioned in discussion.
5. Methods: Many of the model inputs are quite old now, and searches only run until Dec 2019. We don’t propose updating them at this stage, as this would be a large undertaking, but this is a limitation, which should be acknowledged more clearly in the methods section and discussion (it is mentioned briefly in discussion).
6. Table 1: Intervention/Comparator. Can you be clearer when switching occurs between treatments in the model? Do a proportion of patients switch immediately due to intolerance, or is there a switching proportion over time?
7. Table 1 footnote: Reference is to the old NICE methods manual. Update to the 2022 version.
8. Methods_Model Structure subsection: There is just one sentence to refer the reader to the decision tree, and no further explanation given. Please add in a bit of text explaining the paths through the decision tree, especially the treatment switching pathways. Why is there an MRD+ASP path on the Universal clopidogrel tree. Due to intolerance to clopidogrel? What are the timings between the different steps along the pathway in the decision tree? Was the decision tree run for the first cycle i.e. 1 year? Or shorter? The reader can’t be expected to know all this from the tree and text provided.
9. Figure 1: Define the abbreviations for treatments MRD, ASA.
10. Figure 2: The health states are labelled as ‘No further stroke’ in the figure but called ‘No additional stroke’ in the text. It would be good to use the same language for consistency here.
11. Model Assumptions: Some of these are listed, but many are not. For example we think it is assumed that the test is 100% sensitive and 100% specific. Also it’s assumed that all those with a test result will receive the appropriate treatment.
12. Markov Model: Again the description of the model is very brief. Explain why you chose those particular states for the model (e.g. choice of one further and > 1 further stroke). How were bleeding events /ICH captured in the model?
13. P.6 sentence before Populating the Model section: Is this a typo and should read “… treated as atherothrombotic (and treated with antiplatelets) as opposed to …”.
14. Populating the Model: A table that lists all model parameters and assumed input values and distributions for PSA, and evidence sources that they are based upon is missing. Later you refer to “Appendix 1”, so maybe you have prepared this table, but we couldn’t find this appendix.
15. Populating the model_Probabilities subsection, end of first para: “It is assumed that patients switched to MRD+ASA or ASA have similar levels of secondary stroke to clopidogrel in the following the current prescribing pathway strategy”. Are you assuming that in LoF patients on MRD+ASA they have the same probability of stroke as for NoLoF patients on Clopidogrel? This seems like a strong assumption...if it were the case, then why not give everyone MRD+ASA in the first place? Also, it sounds like you are assuming equal efficacy for MRD+ASA and ASA mono, which again doesn’t seem reasonable to me. Can you clarify the assumptions and justify them, providing the evidence sources that support the assumptions? In general the second half of this paragraph is unclear.
16. Resource Use and Costs section/Table 2: Comment on the test costs - is the cost listed per patient? Does that depend on the population size and life-time of the device?
17. Resource Use and Costs: Highlight here the limitation that you are using old costs for health states that will likely be out of date. It would be useful in the table to have the original price year of all the costs in table 2, as not all costs are derived from TA90. Although the costs were inflated to 2021 £ there may some costs which have changed. It would be of particular interest to have the price year used for the cost of the PoC tests.
18. Health consequences: I don’t think you include carer disutility, which is relevant for stroke patients. This should be acknowledged as a limitation in discussion.
19. Data Analysis section: This is fine but quite lengthy. If short on words, it would be better to use them to describe the model in more detail.
20. Given the large number of parameters and assumptions, threshold analysis is only reported for 3 parameters: RR for clopidogrel in No LoF vs general population; test cost; and clopidogrel intolerance. It would be good to see something like a tornado plot showing the ICER ranges for all parameters in the model. Also, any other assumptions that could be varied in scenario analyses e.g. diagnostic accuracy, uptake of targeted treatment.
21. Results: It is reported that "changing the assumed tolerance to taking modified-release dipyridamole did not impact the absolute cost-effectiveness of the intervention at any assigned level”. These results may be in the missing Appendix 1, but if not, it would be useful to be able to see these numbers reported.
22. Discussion 2 ^nd^ para: It’s unclear which figures here are assumptions, which are based on evidence sources, and which are results from the model. E.g. effectiveness of clopidogrel universally has similar effectiveness on average to Dipyridamole. Is there a reference supporting that? Or is it a model output?
23. Discussion para 3: Ticagrelor - should note that this is not currently available in the UK NHS.
24. Discussion para 6: Assumptions made. Also assumed perfect diagnostic test accuracy, 100% uptake of targeted treatment, % with LoF, relative risk of stroke on different treatments/LoF status, etc.
25. Figure 3: There appears to be a strong correlation between the incremental benefits and incremental costs in the cost-effectiveness plane. It would be interesting to hear the authors' interpretation of this result and what model mechanism(s) may be driving it in the discussion.
26. Conclusion, last sentence: I’m not sure I agree with this conclusion, as there has been a large trial of a guided therapy (ticagrelor vs clopidogrel) for secondary prevention of stroke in LoF patients (CHANCE-2, Wang *et al.* 2021), plus a trial of clopidogrel vs aspirin with results by LoF status (CHANCE). Together with sensitivity and specificity of the test, and the model, that may be sufficient. The problem is that there isn’t any evidence on dipyridamole vs clopidogrel in a LoF population, i.e. as in the UK context. I think you could reword this to be more specific to the evidence needs for the UK context.
27. Although this is only an early economic model you may want to complete the Drummond *et al.* CHEERS checklist for health economic evaluations in the Appendix. Many of the checklist items seem to be included in the manuscript.

Is the work clearly and accurately presented and does it cite the current literature?

Yes

If applicable, is the statistical analysis and its interpretation appropriate?

Not applicable

Are all the source data underlying the results available to ensure full reproducibility?

Partly

Is the study design appropriate and is the work technically sound?

Yes

Are the conclusions drawn adequately supported by the results?

Yes

Are sufficient details of methods and analysis provided to allow replication by others?

Partly

Reviewer Expertise:

Health Economic Modelling; evidence synthesis

We confirm that we have read this submission and believe that we have an appropriate level of expertise to confirm that it is of an acceptable scientific standard, however we have significant reservations, as outlined above.
